# Circular RNA NFIX Functions as an Oncogene in Non‐Small Cell Lung Cancer by Modulating the miR‐214‐3p/TRIAP1 Axis

**DOI:** 10.1111/crj.13801

**Published:** 2024-08-12

**Authors:** Guohua Liu, Hanbing Shi, Hongyan Zheng, Weili Kong, Xinyue Cheng, Liling Deng

**Affiliations:** ^1^ Department of Respiratory and Critical Care Medicine The Third Affiliated Hospital of Qiqihar Medical College Qiqihar China; ^2^ Department of Pediatrics The Third Affiliated Hospital of Qiqihar Medical College Qiqihar China

**Keywords:** circRNA NFIX, miRNA‐214‐3p, NSCLC, TRIAP1

## Abstract

**Background:**

circRNA NFIX has been shown to exist as an oncogene in glioma. But its expression and role in NSCLC (non‐small cell lung cancer) are still unclear. This research aimed to discover the expression and function of circRNA NFIX in NSCLC.

**Methods:**

In this research, qRT‐PCR was utilized to investigate the expression levels of circRNA NFIX, miRNA‐214‐3p, and TRIAP1 in NSCLC tissues and cell lines. The binding sites between circRNA NFIX/TRIAP1 and miRNA‐214‐3p were predicted using the Starbase. These interactions were further validated using a double luciferase reporter assay. Cell proliferation and apoptosis were assessed through MTT and flow cytometry, respectively. The expression of apoptosis‐related proteins was measured by western blot assay.

**Results:**

miRNA‐214‐3p could link with circRNA NFIX. circRNA NFIX was upregulated, while miRNA‐214‐3p was downregulated in NSCLC cell lines and clinical samples. Besides, suppression of circRNA NFIX repressed cell proliferation and induced apoptosis in NSCLC cells by upregulating miRNA‐214‐3p expression. Besides, the data indicated that TRIAP1 was a target of miRNA‐214‐3p, and it was negatively regulated by miRNA‐214‐3p in NSCLC cells. The excessive expression of miRNA‐214‐3p suppressed NSCLC cell proliferation and increased apoptosis. In addition, overexpression of TRIAP1 significantly reversed the effects on NSCLC cells caused by miRNA‐214‐3p mimic.

**Conclusion:**

circRNA NFIX silencing repressed the proliferation of NSCLC cells and induced cell apoptosis by regulating the miR‐214‐3p/TRIAP1 axis, which was a potential diagnostic and therapeutic target for NSCLC.

## Graphical abstract

The present study aimed to discover the function of circRNA NFIX in NSCLC. Results revealed that circRNA NFIX silencing repressed the growth of cancer cells and induced cell apoptosis by upregulating miR‐214‐3p and inhibiting TRIAP1 expression, which was a potential biomarker for NSCLC.

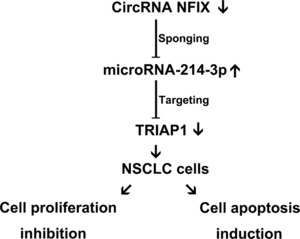

## 1 Introduction

Lung cancer was split into small cell lung cancer (SCLC) and non‐SCLC (NSCLC) [1]. NSCLC was more common than SCLC. NSCLC was less responsive to chemotherapy and radiotherapy when compared to SCLC [2]. For early resectable lung cancer, surgery or surgery combined with chemotherapy may cure some patients. For the majority of patients who could not be surgically resected, radiotherapy provides local control for the majority, and a few of these patients had a chance of cure. Patients in locally advanced stages might also survive long term with a combination of radiotherapy and chemotherapy [3]. Advanced metastatic disease, suitable for chemotherapy, targeted drugs, and supportive therapy, so as to achieve the purpose of improving survival and relieving symptoms. Over the past 15 years, the treatment of NSCLC had developed rapidly. This improvement was mainly due to genomics analysis and the development of targeted therapies [4].

Circular RNAs (circRNAs) are a type of RNA that cannot be translated and have been shown to play a significant role in regulating tumor‐related functions such as metastasis, ferroptosis, and proliferation [5, 6, 7, 8]. circRNA ITCH has been reported to be a potential target for hepatocellular carcinoma [9]. circRNA ARHGEF28 could repress prostate cancer through the miRNA‐671‐5p/LGALS3BP axis [10]. Inhibition of thyroid cancer was regulated by circRNA 0051428 [11]. circRNA NFIX was a novel circRNA that had been indicated to regulate cardiac regeneration [12]. Other research has found that circRNA NFIX plays a critical role in the protective effect of carvedilol on acute myocardial infarction [13]. Although the involvement of circRNA NFIX in various diseases has been documented, its specific role in NSCLC remains unclear. Therefore, we aim to investigate the role and underlying mechanism of circRNA NFIX in NSCLC.

MicroRNA (miRNA) is a class of small RNAs known for its conservative nature, typically spanning around 19 base pairs [14]. Research suggests that numerous miRNAs participate in the development of certain diseases by modulating posttranslational processes [14, 15]. Recent studies on miRNAs in NSCLC have highlighted the significant role of specific miRNAs, including miRNA‐490‐3p [16], miRNA‐196b‐5p [17], and miRNA‐130a [18]. Additionally, miRNA‐214‐3p has been implicated in liver cancer [19]. A recent study indicated that miRNA‐214‐3p was downregulated in NSCLC cell lines [20]. However, the exact function of miRNA‐214‐3p and its underlying mechanisms in NSCLC remain to be elucidated.

TP53 regulation of apoptosis inhibitor 1 (TRIAP1) is regulated by the TP53 evolutionary conserved proteins. TRIAP1 is considered an oncogene because it prevents cancer cell apoptosis by inhibiting the interaction between cytochrome c and apoptotic protease activating factor 1 [21, 22, 23]. Previous research has shown that TRIAP1 is linked to various human diseases, such as colon cancer [24], gestational diabetes mellitus [25], and penile carcinoma [26]. Hao et al. reported that TRIAP1 knockdown sensitizes NSCLC to ionizing radiation by disrupting redox homeostasis [21]. However, the function of TRIAP1 in NSCLC has not been explained clearly.

The purpose of this research is to investigate the role and underlying mechanism of circRNA NFIX in NSCLC.

## 2 Methods and Materials

### 2.1 Clinical Sample Collection

Thirty clinical tumor samples and the corresponding number of paracancer tissues were collected from hospitals and stored in an ultralow temperature (−80°C) refrigerator for subsequent detection. Sample inclusion criteria were as follows: (i) clinically confirmed NSCLC and (ii) had not received any treatment prior to surgery. This study was approved by the Ethics Committee of the Third Affiliated Hospital of Qiqihar Medical College. Every patient provided written informed consent.

### 2.2 Cell Culture

The NSCLC cancer cell lines (A549 and NCI‐H23), normal lung epithelial cell line (BEAS2B), and 293T cells were obtained from Procell (Wuhan, China). They were maintained in F12K medium (Vivacell, China) with the addition of 1% P/S (penicillin and streptomycin) (Vivacell, China) and 10% FBS (Vivacell, China) under a 5% CO_2_ atmosphere at 37°C.

### 2.3 Bioinformatics Information

Starbase (Http://starbase.sysu.edu.cn/index.php) was used to predict the binding sites of miRNA‐214‐3p on circRNA NFIX/TRIAP1.

### 2.4 Dual‐Luciferase Reporter Assay

The 3′‐UTR of circRNA NFIX, along with the mutant circRNA NFIX 3′‐UTR sequence and TRIAP1, was cloned into a pGL‐U6‐puromycin vector (Tsingke, China) for luciferase activity analysis. Next, 293T cells were cotransfected with wild‐type pGL3‐circRNA NFIX (or TRIAP1)‐3′UTR or mutant pGL3‐circRNA NFIX (or TRIAP1)‐3′UTR, along with mimic control and miRNA‐214‐3p mimic, using jetMESSENGER transfection reagent as per the manufacturer's instructions (Polyplus, France). Luciferase activity was measured using a reporter system 48 h posttransfection (Promega, USA).

### 2.5 Cell Transfection

To regulate the level of miRNA‐214‐3p, an inhibitor of miRNA‐214‐3p and an inhibitor control (miRNA‐214‐3p inhibitor: 5′‐ACTGCCTGTCTGTGCCTGCTGT‐3′ and inhibitor control: 5′‐CAGTACTTTTGTGTAGTACAA‐3′), as well as a mimic of miRNA‐214‐3p (sense: 5′‐ACAGCAGGCACAGACAGGCAGU‐3′, antisense: 5′‐UGCCUGUCUGUGCCUGCUGUUU‐3′) and a mimic control (sense: 5′‐UUCUGCCGAAUGUCACGUTT‐3′, antisense: 5′‐ACGUUGCUUGUUCGGAGAATT‐3′), were procured from Sangon (Shanghai, China). To reduce the level of circRNA NFIX, siRNA for NFIX (si‐NFIX: 5′‐GTGACAGAGCTGGTGAGAGTA‐3′) was employed, along with control‐siRNA: 5′‐CACGATAAGACTTTAATGTATTT‐3′ purchased from Thermo Fisher (Fermantas, Lithuania). TRIAP1‐plasmid and the control‐plasmid were sourced from Santa Cruz (USA). These sequences were transfected into cells at 65% confluence using jetMESSENGER (Polyplus, France). Cells were harvested after 48 h of culture at 37°C with 5% CO_2_ following transfection.

### 2.6 Quantitative Reverse Transcription‐Polymerase Chain Reaction (qRT‐PCR)

TRIzol was employed to extract the RNA, and spectrophotometry was implemented to detect the RNA's concentration and purity. Using the NovoScript Plus All‐in‐one 1st Strand cDNA Synthesis SuperMix (gDNA Purge) kit (Novoprotein, China), the RNA was reverse‐transcribed to cDNA. On a StepOneTM Real‐Time PCR machine (Thermo Fisher, USA), the cDNA was expanded with the assistance of the NovoStart SYBR qPCR SuperMix Plus reagent (Novoprotein, China). Denaturation was carried out for 1 min at 95°C, followed by 35–45 cycles of 20 s at 95°C, 20 s at 60°C, and 30 s at 72°C in the qRT‐PCR software. The internal reference was β‐actin or U6. Utilizing the comparative threshold cycle (2^–ΔΔCT^) approach, the relative gene expression gene was determined. Table 1 displays the primer sequences of PCR.

**TABLE 1:** Primer sequences for PCR.

Gene	Primer (5′‐3′)
circRNA NFIX	Sense: TCTCCTACACCTGGTTCAA
	Antisense: CAATGTGATGTGGCTGGA
miRNA‐214‐3p	Sense: GAGTGTTGGCCTGTCCTCAA
	Antisense: TTGTGCCCAGTTGCCTGTAT
TRIAP1	Sense: CGCTGGTTCGCCGAGAAATTTC
	Antisense: TGAAGGACTGGAGTTCATGGGC
β‐actin	Sense: CCATCGCCAGTTGCCGATCC
	Antisense: GCGAGAGGAGCACAGATACCACCAA
U6	Sense: GCTTCGGCAGCACATATACTAAAAT
	Antisense: CGCTTCACGAATTTGCGTGTCAT

### 2.7 Cell Apoptosis Detection

The cells underwent trypsinization, centrifugated, and then washed twice with cold PBS (Solarbio, Beijing). Following resuspension in 100 μL of 1 × Annexin V binding solution (Biolegend, USA), 5 μL of Annexin V‐FITC staining solution (Biolegend, USA) was applied. Next, after gently mixing the cells, they underwent incubation in a dark environment under a condition of 4°C with a duration of 10 min, following the manufacturer's instructions. After that, after applying and cautiously mixing 5 μL of PI staining solution (Biolegend, USA), the mixed solution was cultured for 3 min under a condition of 4°C in the dark. The 1 × Annexin V conjugate solution was then applied and carefully stirred. The cells were immediately assessed by flow cytometry after being run through a 300‐mesh nylon mesh, and FlowJo 10.0 software was used to analyze the cells for analysis.

### 2.8 Cell Proliferation Detection

In 96‐well plates, cells in the logarithmic growth phase were seeded at a density of 3 × 10^3^/100 μL/well, with three duplicate wells each set. After the incubation, 10 μL of the MTT reagent (Fcmacs, Nanjing) was given to every well. The incubation was performed under a condition of temperature 37°C in the dark for an additional 2 h. We used an ultraviolet spectrophotometer (Synergy HT, BioTek, USA) to evaluate the optical density (OD) values at 570 nm.

### 2.9 Western Blot Analysis

A lysis buffer containing 1 mM phenylmethylsulfonate fluoride (PMSF, Labgic, China) was applied to extract the total protein using RIPA (Beyotime, China). Using a fast preparation kit (Yazy Biomedical, China), following electrophoretically separated samples on 10% SDS‐PAGE gels, the samples were subsequently transferred to PVDF membranes (Merck, USA) at a continuous current of 200 mA for 80 min. The membranes were incubated with primary antibodies against caspase3 (ab44976, Abcam), cleaved‐caspase3 (AF7022, affbiotech), TRIAP1 (15 351–1‐AP, Wuhan Sanying), and GAPDH (ab181602, Abcam) for an entire night on a shaking table at 4°C. After that, the membranes were washed with TBST (Solarbio, Beijing) for three times. Subsequently, the membranes were incubated with a secondary antibody for 1 h at the room temperature. After that, the bands were observed using the ECL‐chemiluminescent kit (Biosharp, China) and quantified using ImageJ software from IBM Corp. (Armonk, USA).

### 2.10 Statistical Analysis

SPSS analysis (version 20.0; IBM Corp.) was used for the statistical analysis. The data were presented as the mean ± standard deviation (SD) of three independent measurements. Student's *t*‐test was used to analyze the statistical comparisons among two groups. Statistical comparisons among multiple groups were analyzed by one‐way analysis of variance (ANOVA) followed by Tukey's post hoc test. *p* < 0.05 means a statistically difference.

## 3 Results

### 3.1 Interaction Sites Between circRNA NFIX and miRNA‐214‐3p

**FIGURE 1:**
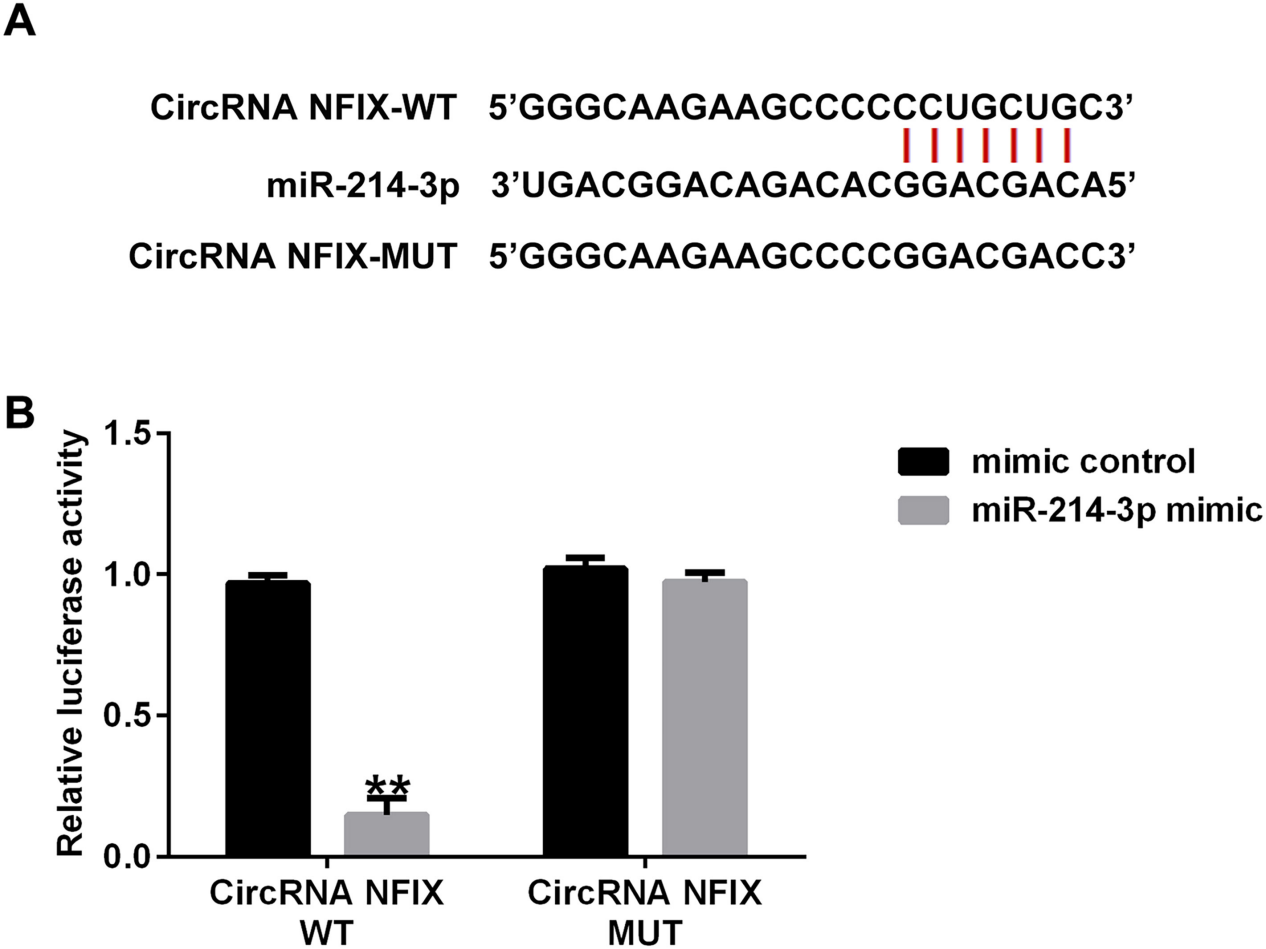
circRNA NFIX targets miRNA‐214‐3p. (A) The binding sites between miRNA‐214‐3p and circRNA NFIX. (B) Dual‐luciferase reporter assay was performed to confirm the binding sites between miRNA‐214‐3p and circRNA NFIX. ** indicates *p* < 0.01. Data are exhibited as average ± SD of triple single experiments.

To investigate the potential binding sites of circRNA NFIX and miRNA‐214‐3p, Starbase was used. The binding sites between miRNA‐214‐3p and circRNA NFIX are shown in Figure 1A. As shown in Figure 1B, compared with the cells cotransfected with circRNA NFIX‐WT and mimic control, the luciferase activity of cells cotransfected with circRNA NFIX‐WT and miRNA‐214‐3p mimic significantly decreased, while no significant changes of the luciferase activity were observed in cells cotransfected with circRNA NFIX‐MUT and mimic control, and cells cotransfected with circRNA NFIX‐MUT and miRNA‐214‐3p mimic. These findings indicated that circRNA NFIX interacts directly with miRNA‐214‐3p.

### 3.2 circRNA NFIX Was Upregulated, and miRNA‐214‐3p Was Downregulated in NSCLC

**FIGURE 2:**
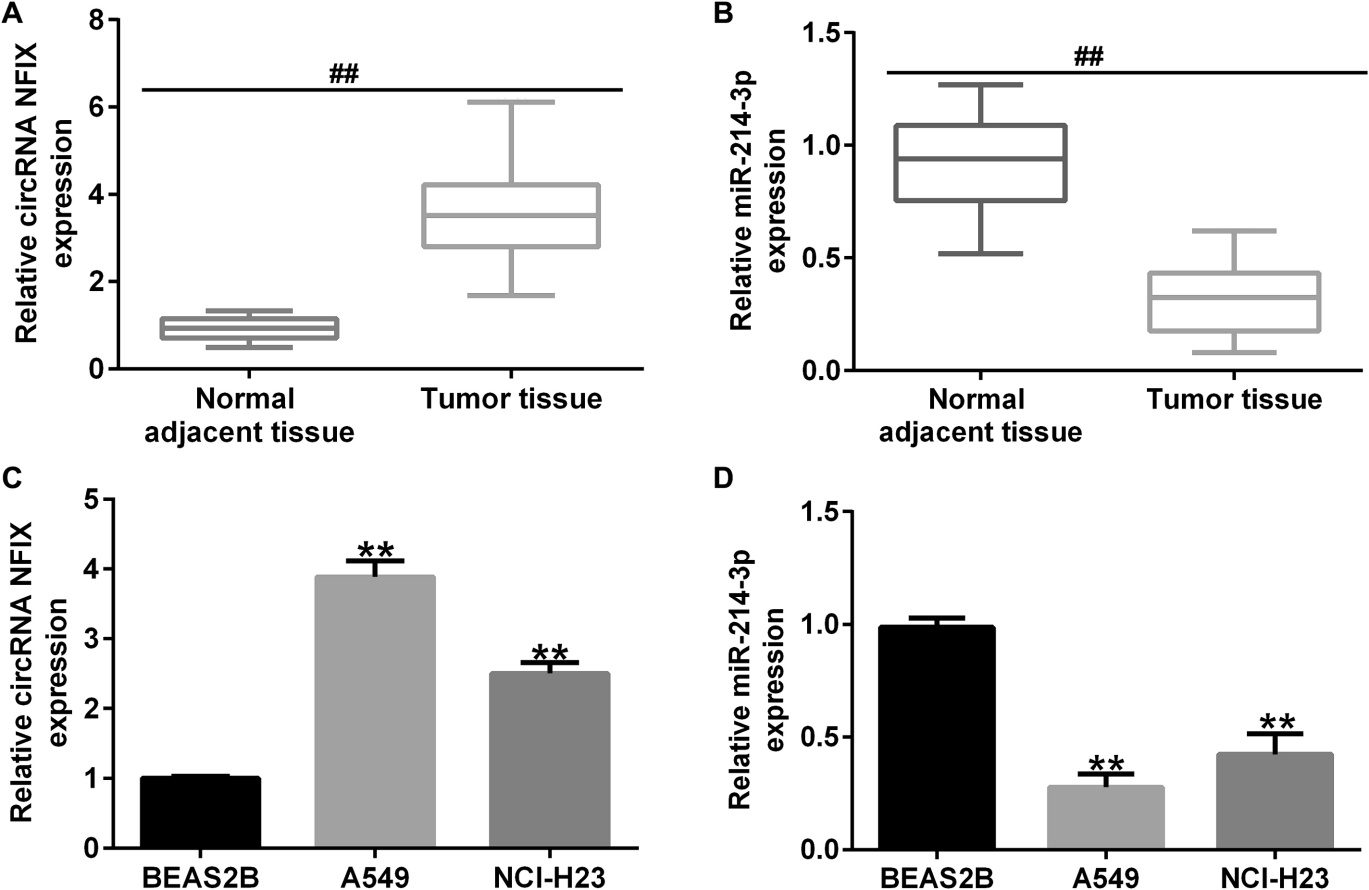
Level of circRNA NFIX and miRNA‐214‐3p in NSCLC. (A and B) The level of circRNA NFIX and miRNA‐214‐3p in NSCLC cancer tissue was detected by qRT‐PCR. (C and D) The level of circRNA NFIX and miRNA‐214‐3p in NSCLC cell lines and in normal lung epithelial cell line (BEAS2B) was detected by qRT‐PCR. ** indicates *p* < 0.01, and ## indicates *p* < 0.01. Data are exhibited as average ± SD of triple single experiments.

In order to determine the expression of circRNA NFIX and miRNA‐214‐3p in NSCLC, qRT‐PCR was used. Results hinted that compared with the normal adjacent tissue, circRNA NFIX was upregulated and miRNA‐214‐3p was downregulated in NSCLC tissue (Figure 2A,B). In comparison to normal lung cells, there was a substantial rise in circRNA NFIX expression in lung cancer cell lines, accompanied by a decrease in miR‐214‐3p levels. Among them, A549 cells demonstrated the most notable alterations (Figure 2C,D). In conclusion, both circRNA NFIX and miRNA‐214‐3p were implicated in the progression of NSCLC.

### 3.3 circRNA NFIX Negatively Regulated the Level of miRNA‐214‐3p in A549 Cells

**FIGURE 3:**
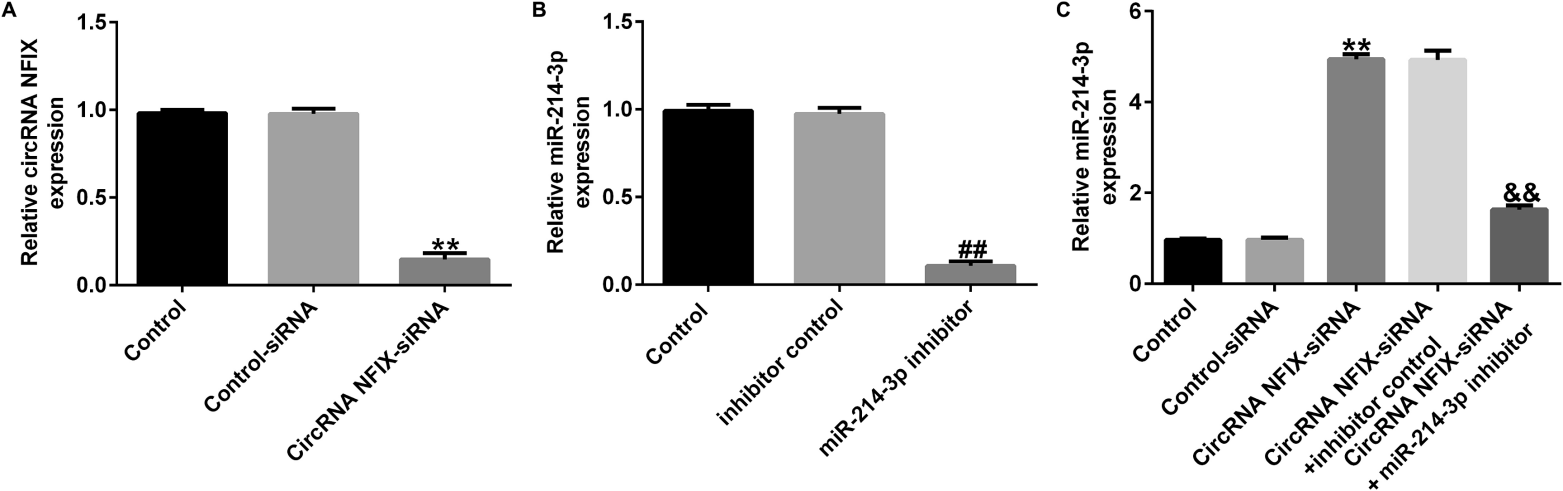
The level of miRNA‐214‐3p was negatively regulated by circRNA NFIX in NSCLC cells. (A) The efficiency of circRNA NFIX‐siRNA transfection in A549 cells was determined by qRT‐PCR. (B) The efficiency of miRNA‐214‐3p inhibitor transfection in A549 cells was determined by qRT‐PCR. (C) The level of miRNA‐214‐3p in A549 cells transfected with circRNA NFIX‐siRNA/miRNA‐214‐3p inhibitor was calculated by qRT‐PCR. ** indicates *p* < 0.01 versus control‐siRNA, ## indicates *p* < 0.01 versus inhibitor control, and && indicates *p* < 0.01 versus circRNA NFIX‐siRNA + inhibitor control. Data are exhibited as average ± SD of triple single experiments.

In order to verify whether circRNA NFIX and miRNA‐214‐3p really exist in direct binding sites, we transfected the A549 cells with circRNA NFIX‐siRNA or miRNA‐214‐3p inhibitor. The transfection efficiency was determined by qRT‐PCR after a period of 48 h. The expression of circRNA NFIX was notably disrupted by circRNA NFIX‐siRNA in A549 cells compared to the control‐siRNA group (Figure 3A). The expression of miR‐214‐3p was significantly reduced in A549 cells by the miR‐214‐3p inhibitor compared to the inhibitor control group (Figure 3B). circRNA NFIX‐siRNA led to a significant increase in miR‐214‐3p expression in A549 cells, which was effectively blocked by cotransfection with the miR‐214‐3p inhibitor (Figure 3C). These findings confirm the presence of direct binding sites between circRNA NFIX and miR‐214‐3p.

### 3.4 circRNA NFIX‐siRNA Inhibited Cell Proliferation and Promoted Apoptosis in Lung Cancer Cells Through the Regulation of miRNA‐214‐3p

**FIGURE 4:**
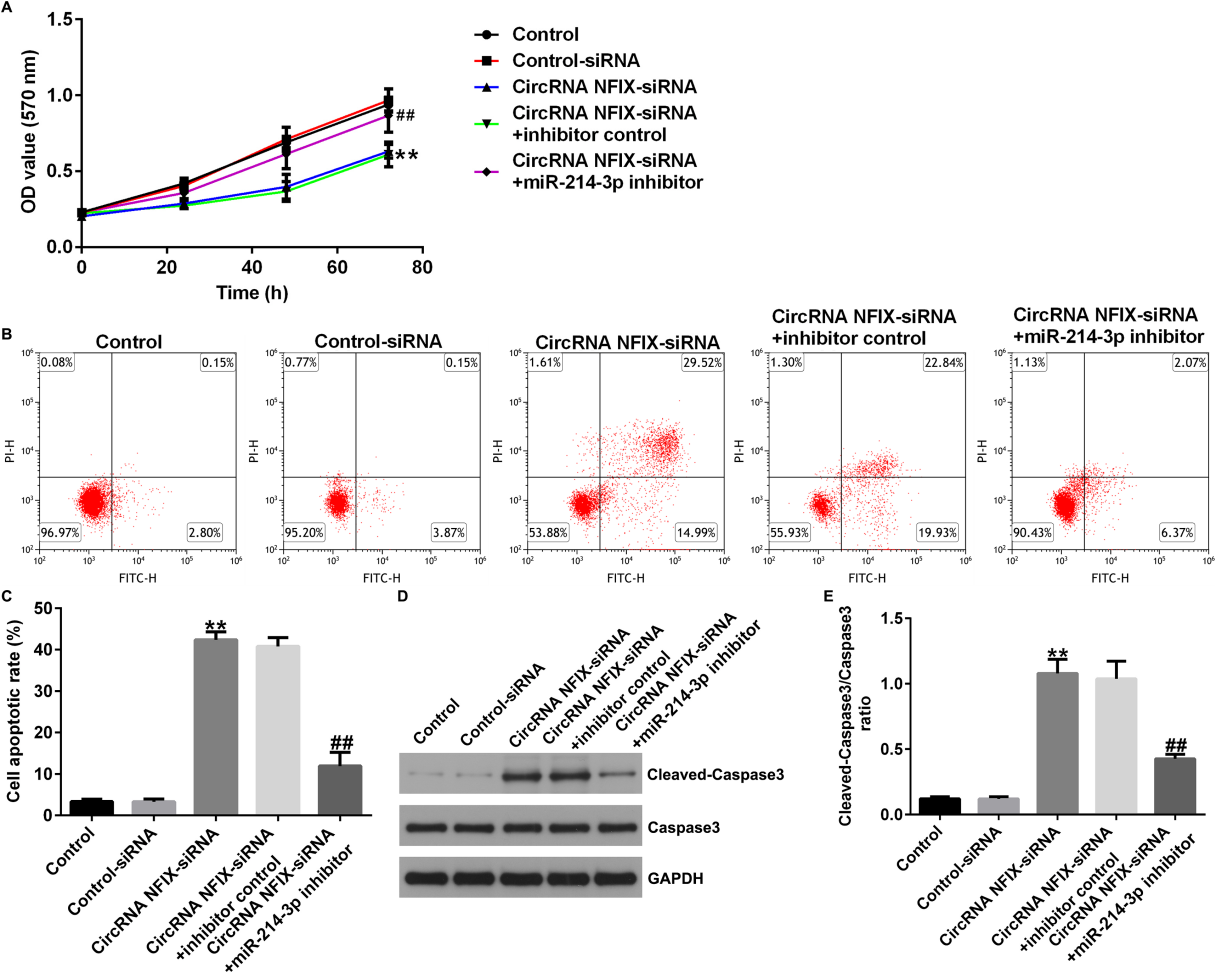
Effect of circRNA NFIX interference in NSCLC by targeting miRNA‐214‐3p. A549 cells were transfected with control‐siRNA, circRNA NFIX‐siRNA, circRNA NFIX‐siRNA + inhibitor control, or circRNA NFIX‐siRNA + miR‐214‐3p inhibitor for 48 h. (A) Cell proliferation was counted by MTT assay. (B and C) The apoptosis ratio of cancer cells was detected by flow cytometry. (D and E) The level of cleaved‐caspase3 was detected by western blot assay, and cleaved‐caspase3/caspase3 ratio was calculated. ** indicates *p* < 0.01 versus control‐siRNA, ## indicates *p* < 0.01 circRNA NFIX‐siRNA + inhibitor control. Data are exhibited as average ± SD of triple single experiments.

A549 cells were transfected with control‐siRNA, circRNA NFIX‐siRNA, circRNA NFIX‐siRNA + inhibitor control, or circRNA NFIX‐siRNA + miR‐214‐3p inhibitor for 48 h. Results indicated that the cell proliferation was significantly reduced in the group transfected with circRNA NFIX‐siRNA compared to the control‐siRNA group, and this reduction was reversed by the miR‐214‐3p inhibitor (Figure 4A). In the circRNA NFIX‐siRNA group, cell apoptosis was significantly increased, and miRNA‐214‐3p inhibitor cotransfection could inhibit cell apoptosis (Figure 4B and C). Accordingly, a western blot assay was conducted to measure the protein level of cleaved‐caspase3 and caspase3; results showed that in the circRNA NFIX‐siRNA transfection group, cleaved‐caspase3 expression and cleaved‐caspase3/caspase3 ratio were also enhanced, and the phenomenon was also repressed by miRNA‐214‐3p inhibitor (Figure 4D,E).

### 3.5 miR‐214‐3p Directly Targeted TRIAP1

**FIGURE 5:**
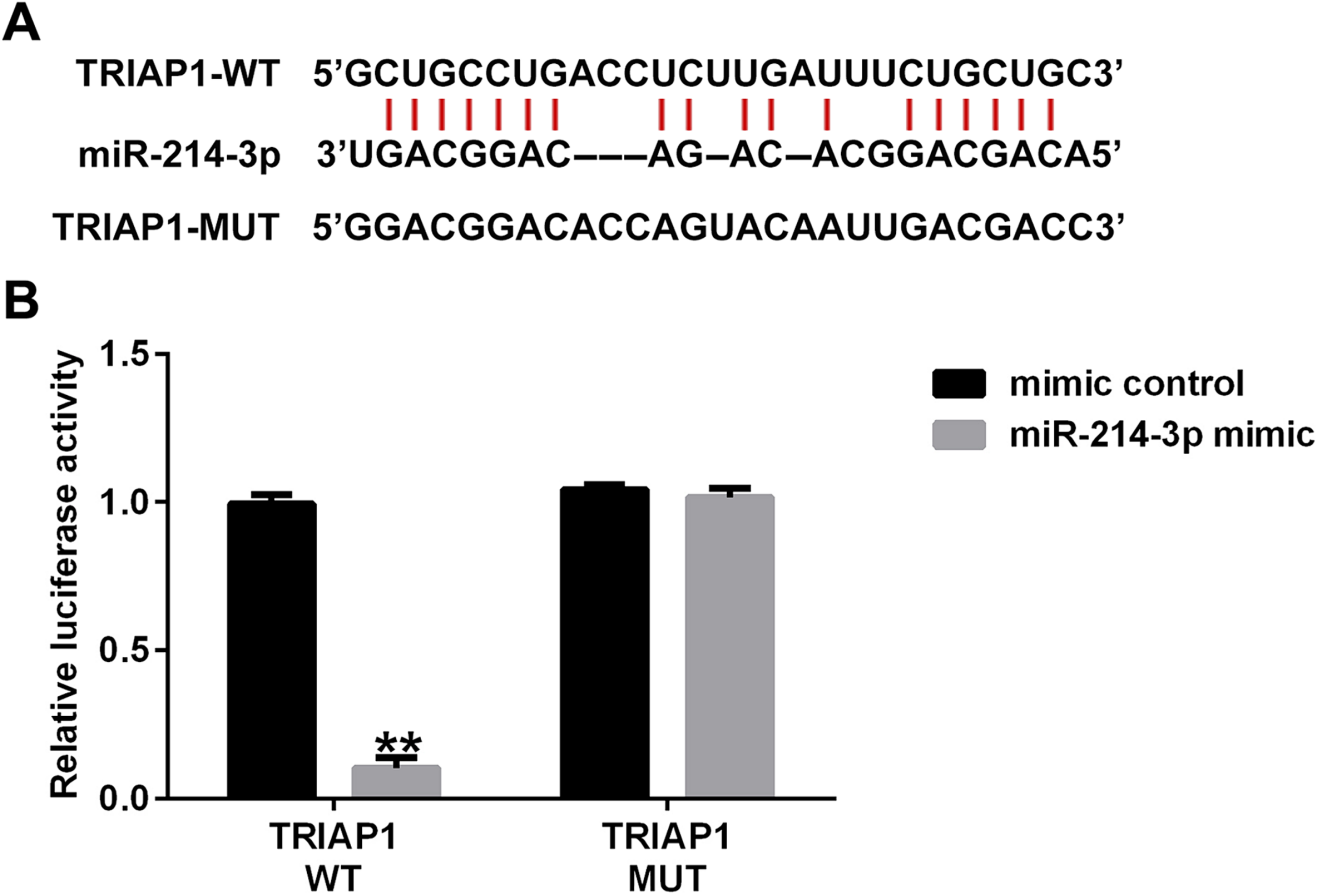
TRIAP1 was a downstream target of miRNA‐214‐3p. (A) The foreknowledge binding points between miRNA‐214‐3p and TRIAP1 were forecasted by Starbase. (B) The dual relative luciferase assay was used for the confirmation of the linkage between miRNA‐214‐3p and TRIAP1. ** indicates *p* < 0.01. Data are exhibited as average ± SD of triple single experiments.

We identified the mRNA target of miRNA‐214‐3p using Starbase, and we discovered that TRIAP1 harbored binding sites for miRNA‐214‐3p (Figure 5A). We utilized the dual luciferase assay to investigate this interaction. The results revealed that the upregulation of miRNA‐214‐3p led to a reduction in the luciferase activity of the TRIAP1‐WT (Figure 5B), indicating that TRIAP1 was a direct target of miRNA‐214‐3p.

### 3.6 miR‐214‐3p Negatively Regulated TRIAP1 Expression in A549 Cells

**FIGURE 6:**
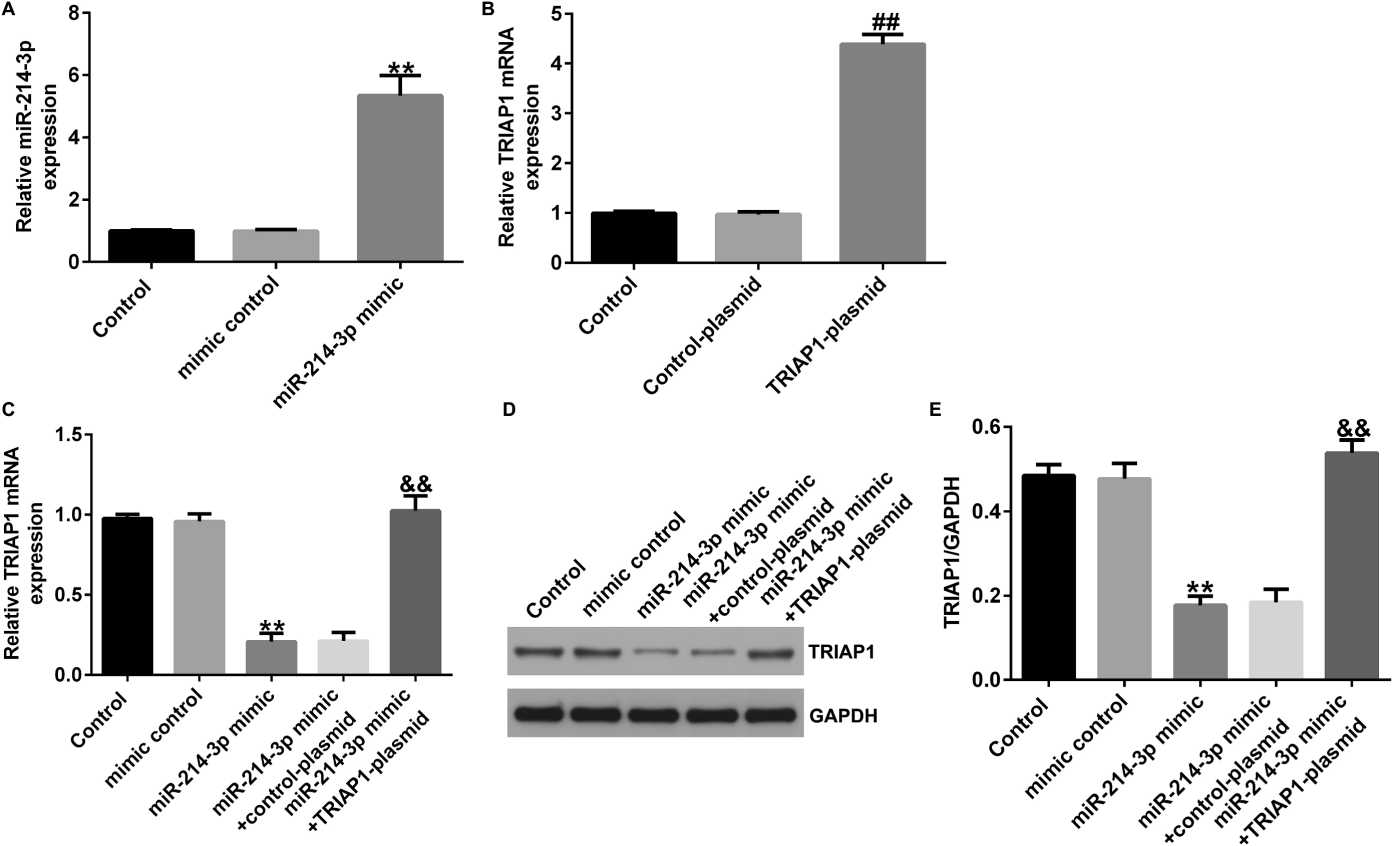
TRIAP1 was negatively controlled by miRNA‐214‐3p in A549 cells. (A) The efficiency of miR‐214‐3p mimic transfection in A549 cells was assessed by qRT‐PCR. (B) The efficiency of TRIAP1‐plasmid transfection in A549 cells was assessed by qRT‐PCR. (C and D) The level of TRIAP1 in A549 cells transfected with mimic control, miRNA‐214‐3p mimic, miRNA‐214‐3p mimic + control‐plasmid, or miRNA‐214‐3p mimic + TRIAP1‐plasmid was counted by qRT‐PCR and western blot assay. ** indicates *p* < 0.01 versus mimic control, ## indicates *p* < 0.01 versus control‐plasmid, and && indicates *p* < 0.01 versus miR‐214‐3p mimic + control‐plasmid. Data are exhibited as average ± SD of triple single experiments.

A549 cells were transfected with mimic control, miRNA‐214‐3p mimic, miRNA‐214‐3p mimic + control‐plasmid, or miRNA‐214‐3p mimic + TRIAP1‐plasmid for 48 h. qRT‐PCR results hinted that miRNA‐214‐3p mimic extremely increased the expression of miRNA‐214‐3p in A549 cells (Figure 6A). In Figure 6B, TRIAP1‐plasmid was found to enhance the mRNA expression of TRIAP1 in A549 cells compared to the control‐plasmid. The introduction of miRNA‐214‐3p mimic led to a notable decrease in TRIAP1 expression in A549 cells, and this change was effectively reversed by cotransfection with TRIAP1‐plasmid (Figure 6C,D). These outcomes indicated that miRNA‐214‐3p negatively regulated TRIAP1 expression in A549 cells.

### 3.7 miRNA‐214‐3p Inhibited the Growth and Induced Apoptosis of A549 Cells by Inhibiting the Expression of TRIAP1

**FIGURE 7:**
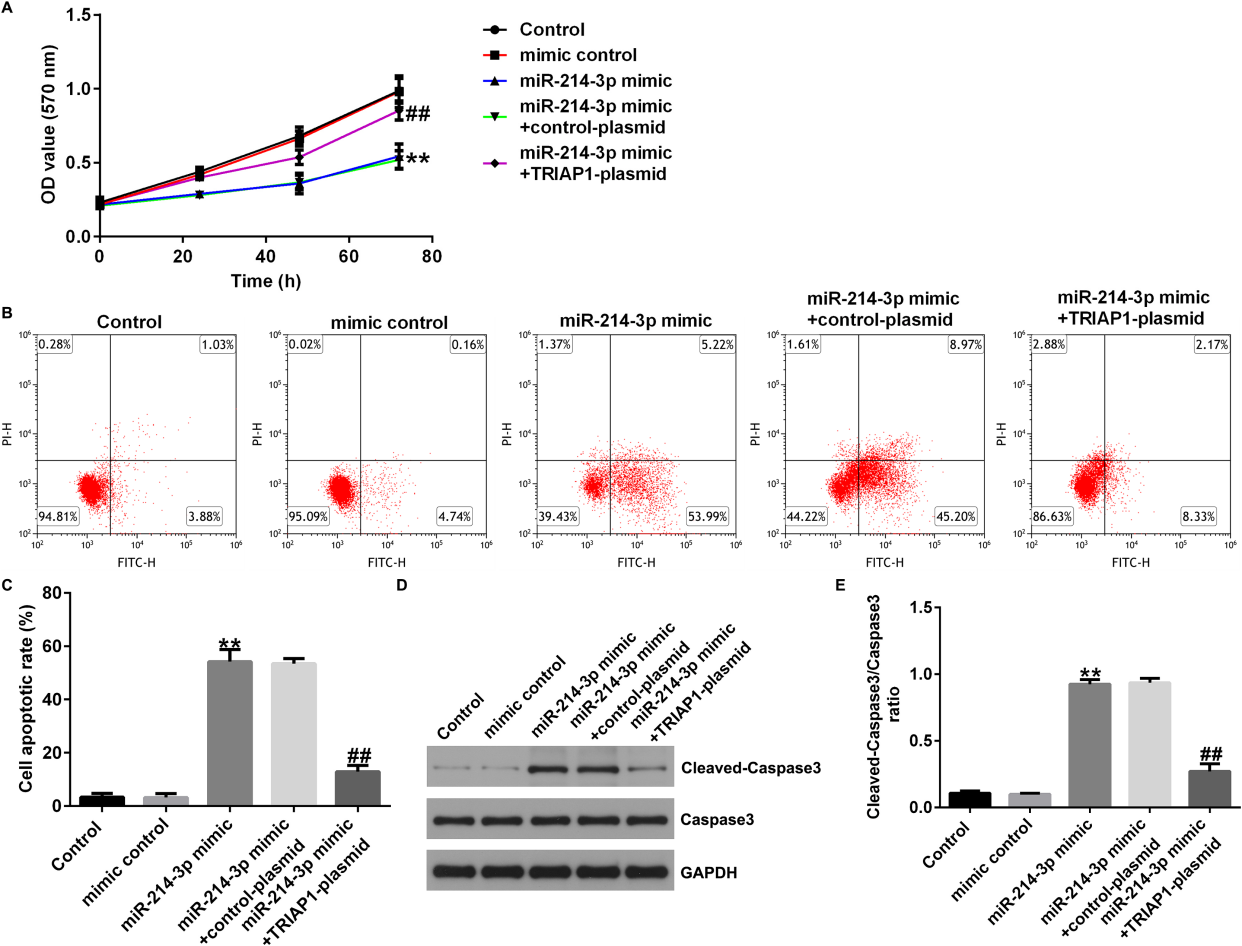
Overexpression of miRNA‐214‐3p suppressed cell growth and promoted cell apoptosis via TRIAP1 in NSCLC cells. A549 cells were transfected with mimic control, miRNA‐214‐3p mimic, miRNA‐214‐3p mimic + control‐plasmid, or miRNA‐214‐3p mimic + TRIAP1‐plasmid for 48 h. (A) Cell proliferation was counted by MTT assay. (B and C) The apoptosis of A549 cells was analyzed by flow cytometry. (D and E) The level of cleaved‐caspase3 was detected by western blot assay, and the ratio of cleaved‐caspase3/caspase3 was determined. ** indicates *p* < 0.01 versus mimic control, ## indicates *p* < 0.01 versus miR‐214‐3p mimic + control‐plasmid. Data are exhibited as average ± SD of triple single experiments.

To observe the effects of TRIAP1 and miRNA‐214‐3p on NSCLC cells, A549 cells were transfected with mimic control, miRNA‐214‐3p mimic, miRNA‐214‐3p mimic + control‐plasmid, or miRNA‐214‐3p mimic + TRIAP1‐plasmid for 48 h. In comparison to the mimic control group, cell proliferation in the miRNA‐214‐3p mimic transfection group was obviously decreased, and TRIAP1‐plasmid significantly reversed this effect (Figure 7A). In the miRNA‐214‐3p mimic transfection group, cell apoptosis was significantly increased, and TRIAP1‐plasmid cotransfection could inhibit cell apoptosis (Figure 7B,C). Accordingly, the expression of cleaved‐caspase3 and cleaved‐caspase3/caspase3 ratio were significantly enhanced by miRNA‐214‐3p mimic, and this phenomenon was also repressed by TRIAP1‐plasmid (Figure 7D,E).

## 4 Discussion

In China, lung cancer has been a prevalent malignant tumor over the last 20 years. A retrospective study on causes of death carried out in the 1970s revealed that the mortality rate of lung cancer was significant, placing it as the fifth most common cancer cause of death after gastric cancer, esophageal cancer, liver cancer, and cervical tumor [27]. Lung cancer accounted for 7.43% of all cancer fatalities. Results from a subsequent survey on causes of death revealed that in the 1990s, lung cancer was the third leading cause of death. A review conducted in the 21st century indicated that lung cancer had become the primary cause of cancer‐related deaths [28]. From a pathology and treatment standpoint, lung cancer can be generally categorized into two groups: NSCLC and SCLC, with the majority falling under NSCLC, accounting for 80%–85% of cases [29]. With the exception of a few early cases, the primary treatment for NSCLC has typically involved a combination of chemotherapy and radiotherapy due to the distinct biological features of the disease.

In recent years, the role of cirRNA in cancer progression, diagnosis, and treatment has been widely studied [30, 31, 32, 33]. Compelling evidence has been uncovered indicating that circRNAs play a crucial role in NSCLC. In NSCLC, kinds of circRNAs were described as biomarkers. circRNA NFIX had been implicated in the development of many other cancers in previous studies. Previous research hinted that temozolomide resistance in glioma was enhanced by circRNA NFIX [34]. Process of acute myeloid leukemia was suppressed by circRNA NFIX interference [35]. Procession of pituitary adenoma was enhanced by circRNA NFIX via CCNB1 [36]. In hepatocellular carcinoma, circRNA NFIX had been reported to be a key regulatory factor and a potential target for hepatocellular carcinoma [37]. Nevertheless, there have been limited studies on the biological roles of circRNA NFIX in NSCLC. We found that circRNA NFIX was significantly elevated in NSCLC. These findings have expanded our current understanding of the involvement of circRNA NFIX in the control of NSCLC, potentially aiding in a more thorough exploration of the pathological mechanisms of NSCLC.

Numerous researchers have discussed how circRNAs regulate disease processes through miRNAs [38]. Jin, Yu, and Zhong hinted that miRNA‐1269a participated in NSCLC through SOX6 [39]. Overexpression of miRNA‐621 and downregulation of SIX4 suppressed the metastasis of NSCLC [40]. In addition, miRNA‐30a was reported that may be a novel biomarker for NSCLC [41]. Experimental evidence of this study revealed that miRNA‐214‐3p functions as a downstream target of circRNA NFIX. miRNA‐214‐3p has been reported to be downregulated in NSCLC cell lines [20]. And the exact function of miRNA‐214‐3p and its underlying mechanisms in NSCLC remains to be elucidated. Thus, miRNA‐214‐3p was selected for further investigation in this study. The data indicated that the expression of miRNA‐214‐3p was significantly reduced in NSCLC. circRNA NFIX knockdown significantly inhibited NSCLC cell proliferation and induced cell apoptosis through upregulating miRNA‐214‐3p expression.

In addition, we found through bioinformatics analysis that miRNA‐214‐3p has many target genes, including TRIAP1. TRIAP1 is participated with the process of many cancers, including osteosarcoma (OS) [42], gastric cancer [43], and ovarian cancer [23]. TRIAP1 is an oncogene that prevents cancer cell apoptosis by inhibiting the interaction between cytochrome c and apoptotic protease activating factor 1. Knockdown of TRIAP1 makes NSCLC sensitive to ionizing radiation [21], indicating that TRIAP1 may play critical roles in regulating cell apoptosis and proliferation in NSCLC. Thus, TRIAP1 was chosen for further study. The findings indicated that miRNA‐214‐3p negatively regulated TRIAP1 expression in NSCLC cells, and miRNA‐214‐3p suppressed NSCLC cell proliferation and induced cell apoptosis by targeting TRIAP1.

There were also some limitations of this study. Firstly, this study only investigated the effect of circRNA NFIX on the proliferation and apoptosis of NSCLC cells. Besides, circRNA NFIX has many target miRNAs, and this study only explored one of them in detail (miR‐214‐3p). In addition, this study is a preliminary in vitro study and no in vivo animal experiments were conducted. In future research, we will further investigate the other effects of circRNA NFIX on NSCLC, including migration, invasion, pyroptosis, and autophagy. We will also further explore other upstream and downstream targets of circRNA NFIX in NSCLC. Most importantly, we will investigate the impact of circRNA NFIX on animal models of NSCLC in our next research.

In summary, our study provided evidence that blocking circRNA NFIX can influence the miRNA‐214‐3p/TRIAP1 pathway to inhibit the progression of NSCLC. Our findings suggest that targeting circRNA NFIX/miRNA‐214‐3p/TRIAP1 could offer a novel approach for treating NSCLC.

## Author Contributions

Guohua Liu contributed to data collection, statistical analysis, data interpretation, and manuscript preparation. Hanbing Shi, Hongyan Zheng, Weili Kong, and Xinyue Cheng contributed to data collection and data interpretation. Liling Deng contributed to statistical analysis and manuscript preparation. All authors have read and approved the final manuscript.

## Ethics Statement

This study was sanctioned by the Ethics Committee of the Third Affiliated Hospital of Qiqihar Medical College.

## Consent

All patients agreed for publication. Written informed consent was obtained from each patient.

## Conflicts of Interest

The authors declare no conflicts of interest.

## Data Availability

The data that support the findings of this study are available from the corresponding author upon reasonable request.
