# Correction to “Circular RNA NFIX Functions as an Oncogene in Non‐Small Cell Lung Cancer by Modulating the miR‐214‐3p/TRIAP1 Axis”

**DOI:** 10.1111/crj.70096

**Published:** 2025-06-19

G. Liu , H. Shi , H. Zheng , W. Kong , X. Cheng , L. Deng , “Circular RNA NFIX Functions as an Oncogene in Non‐Small Cell Lung Cancer by Modulating the miR‐214‐3p/TRIAP1 Axis,” Clinical Respiratory Journal 18, no. 8 (2024). 10.1111/crj.13801.PMC1131908939135128

Figures 4 and 7 are incorrect. Below are the correct figures.

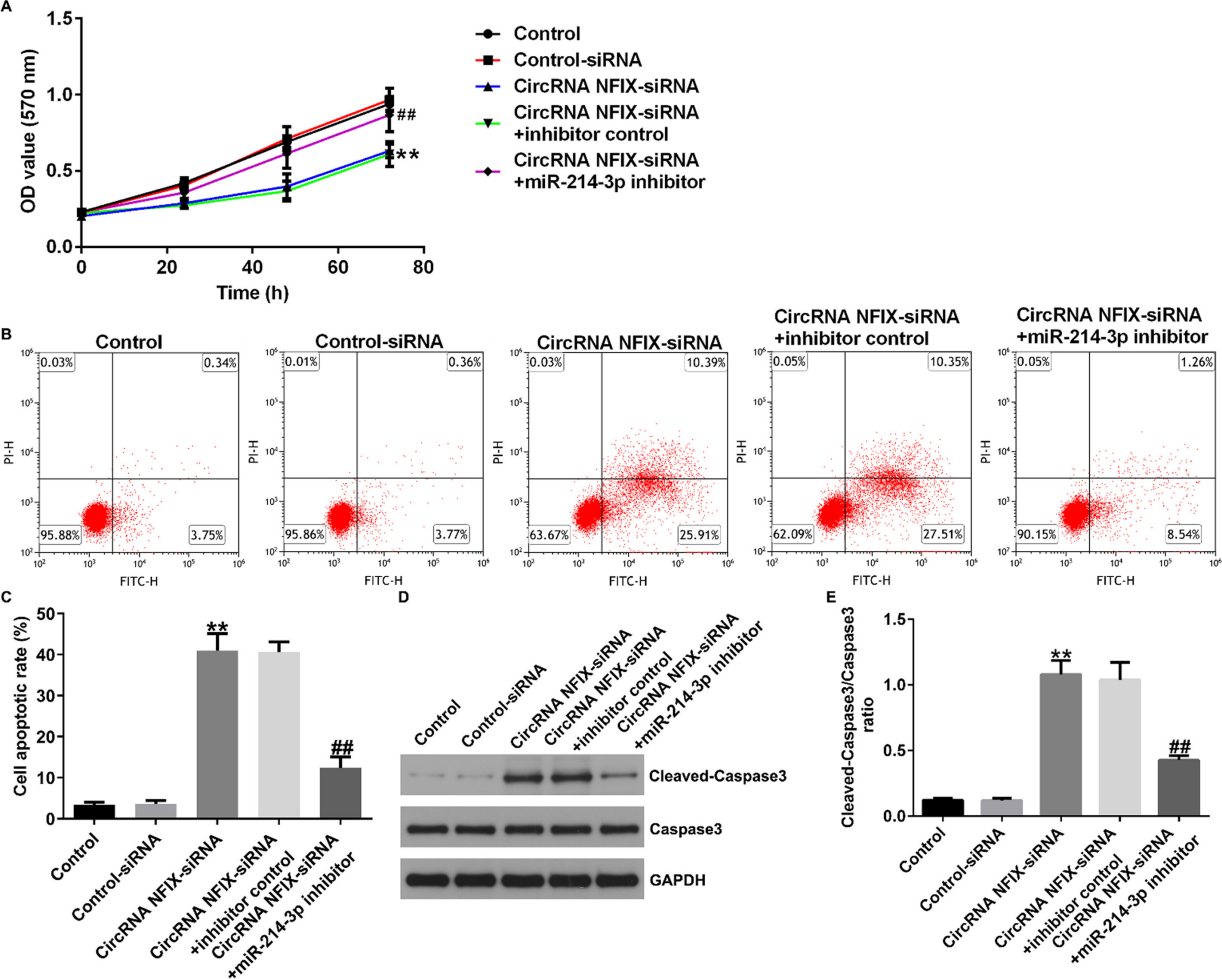

FIGURE 4 | Effect of circRNA NFIX interference in NSCLC by targeting miRNA‐214‐3p. A549 cells were transfected with control‐siRNA, circRNA NFIX‐siRNA, circRNA NFIX‐siRNA + inhibitor control, or circRNA NFIX‐siRNA + miR‐214‐3p inhibitor for 48 h. (A) Cell proliferation was counted by MTT assay. (B and C) The apoptosis ratio of cancer cells was detected by flow cytometry. (D and E) The level of cleaved‐caspase3 was detected by western blot assay, and cleaved‐caspase3/caspase3 ratio was calculated. ** indicates *p* < 0.01 versus control‐siRNA, ## indicates *p* < 0.01 circRNA NFIX‐siRNA + inhibitor control. Data are exhibited as average ± SD of triple single experiments.

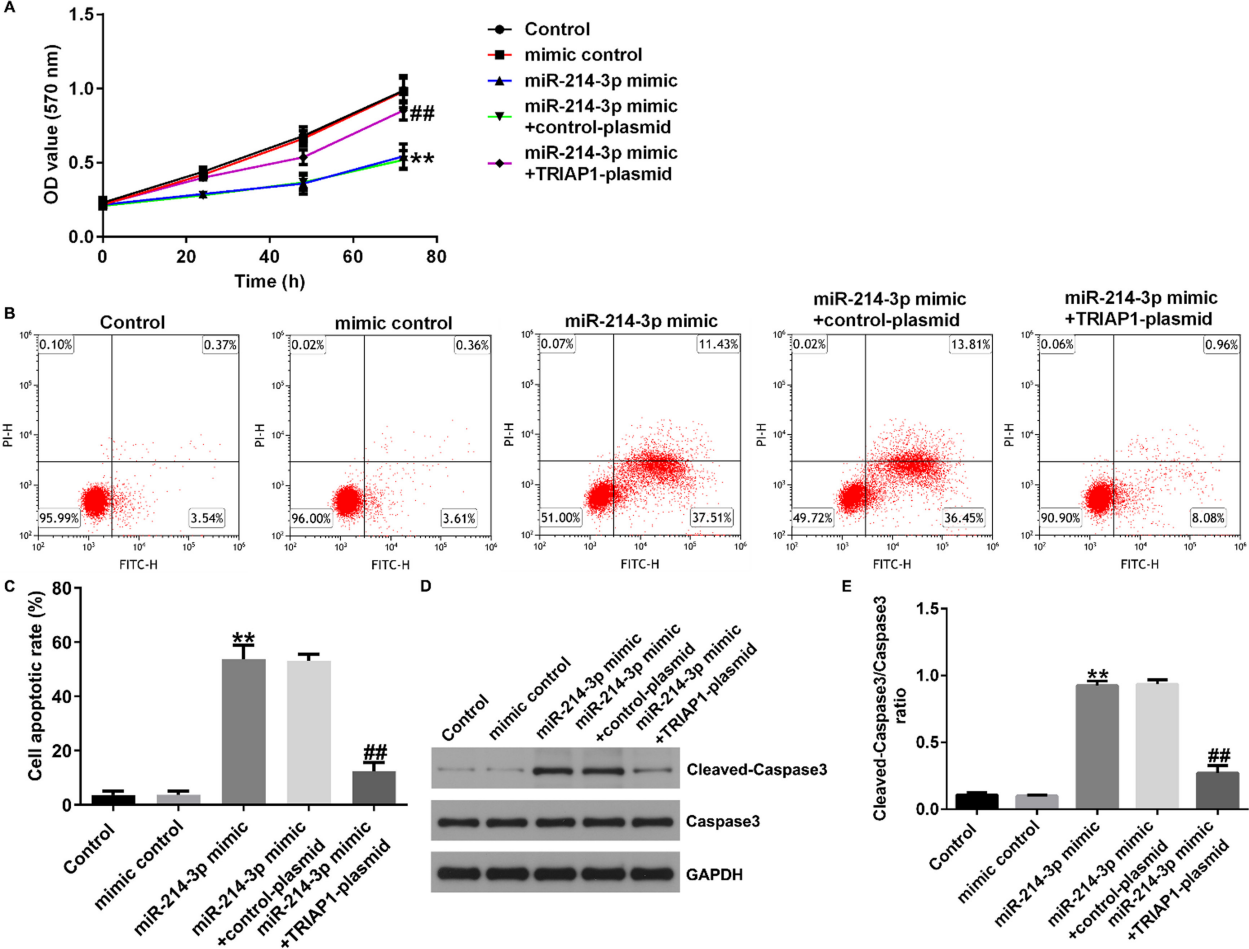

FIGURE 7 | Overexpression of miRNA‐214‐3p suppressed cell growth and promoted cell apoptosis via TRIAP1 in NSCLC cells. A549 cells were transfected with mimic control, miRNA‐214‐3p mimic, miRNA‐214‐3p mimic + control‐plasmid, or miRNA‐214‐3p mimic + TRIAP1‐plasmid for 48 h. (A) Cell proliferation was counted by MTT assay. (B and C) The apoptosis of A549 cells was analyzed by flow cytometry. (D and E) The level of cleaved‐caspase3 was detected by western blot assay, and the ratio of cleaved‐caspase3/caspase3 was determined. ** indicates *p* < 0.01 versus mimic control, ## indicates *p* < 0.01 versus miR‐214‐3p mimic + control‐plasmid. Data are exhibited as average ± SD of triple single experiments.

We apologize for these errors.

